# Breach rhythm-induced asymmetric post-arousal hypersynchrony mimicking ictal EEG in coma

**DOI:** 10.1016/j.cnp.2025.11.001

**Published:** 2025-11-06

**Authors:** Philippe GELISSE, Arielle CRESPEL

**Affiliations:** a Epilepsy Unit, Hôpital Gui de Chauliac, Montpellier, France; b Research Unit (URCMA: Unité de Recherche sur les Comportements et Mouvements Anormaux), INSERM, U661, Montpellier F-34000, France

**Keywords:** SIRPIDs, Sleep-wake transition, Arousal, Coma, Nonconvulsive status epilepticus, ACNS terminology, Salzburg criteria

## Abstract

**Objective:**

To highlight the risk of misinterpreting stimulation-related EEG patterns as seizures in the ICU, and to illustrate how awakening hypersynchrony and breach rhythm can mimic a focal seizure, leading to an erroneous diagnosis of nonconvulsive status epilepticus (NCSE).

**Method:**

A single patient case observation of a 17-year-old male with a severe traumatic brain injury, resulting in a left depressed skull fracture and coma. A continuous EEG was analyzed during repeated auditory/somatosensory stimulations. Pharmacologic reactivity was assessed with IV clonazepam.

**Results:**

Several episodes of awakening hypersynchrony were initially misinterpreted as subclinical focal seizures, leading to a diagnosis of NCSE. The EEGs displayed asymmetrical, sharply contoured rhythmic delta waves after stimulations, showing evolution and lasting more than 10 s. These delta waves demonstrated reactivity to IV-clonazepam, resulting in an improved EEG. Although no clinical improvement was observed, the patient returned to sleep immediately. The EEGs were reinterpreted as representing arousal reactions during the awakening process. The asymmetry of the rhythmic delta waves corresponded to a breach rhythm.

**Conclusion:**

In comatose or sedated ICU patients, stimulation-induced rhythmic delta waves with apparent evolution, and even benzodiazepine responsiveness, may represent normal arousal phenomena amplified by a breach rhythm rather than ictal activity. **Significance:** As stimulus-induced rhythmic, periodic, or ictal discharges (SIRPIDs) strictly describe EEG morphology and evolution without reference to the cause, normal arousals/awakenings and their variations must be recognized in comatose patients. Due to the ambiguity of the term “ictal” and its clinical implications, “ictal” could be replaced with “intermittent”: Stimulus-Induced Rhythmic or Periodic Intermittent Discharges.

## Highlights

• SIRPIDs are rhythmic, periodic, or ictal discharges triggered by alerting stimuli.
• The word “ictal” in the acronym complicates the interpretation of EEGs.
• In coma, identify physiologic arousals to avoid misreading these patterns as seizures.

## 1 Introduction

The effect of somatosensory stimulation on EEGs in comatose patients has been documented for over 50 years (Chatrian et al., 1963, Schwartz and Scott, 1978). To describe the occurrence of rhythmic and periodic patterns, or even ictal-appearing discharges or seizures, Hirsch et al. (2004) proposed the term stimulus-induced rhythmic, periodic, or ictal discharges (SIRPIDs) (Hirsch et al., 2004). This acronym, despite being endorsed by electroencephalographers, sparked intense debate over whether SIRPIDs are associated with seizures or constitute seizures themselves. Terminology published by the American Clinical Neurophysiology Society sought to remove “ictal” as a descriptor of stimulus-induced (SI-) patterns. Rather than using the general term SIRPIDs, the specific SI-pattern is the point of reference (Fong and Hirsch, 2024).

Rhythmic delta activities consist of repeated waveforms exhibiting a fairly uniform morphology and duration, without breaks between consecutive waveforms. If the SI pattern is generalized, the presence of generalized rhythmic delta activity (GRDA) alone does not increase the likelihood of electrographic seizures or status epilepticus. In contrast, if the SI-pattern is lateralized rhythmic delta activity (LRDA), there are strong associations with seizures (Fong and Hirsch, 2024). The significance of SI-LRDA is similar to that of spontaneous LDRA (Fong and Hirsch, 2024). Additionally, LRDA that evolves in frequency, morphology, or location and lasts for at least 10 s may also qualify as electrographic seizures (Hirsch et al., 2023, Hirsch et al., 2021).

We report the case of an adolescent who had several episodes of awakening hypersynchrony with asymmetrical rhythmic delta waves due to a breach rhythm that were initially misinterpreted as focal electrographic seizures, leading to an incorrect diagnosis of nonconvulsive status epilepticus (NCSE).

## 2 Case report

**Fig. 1:**
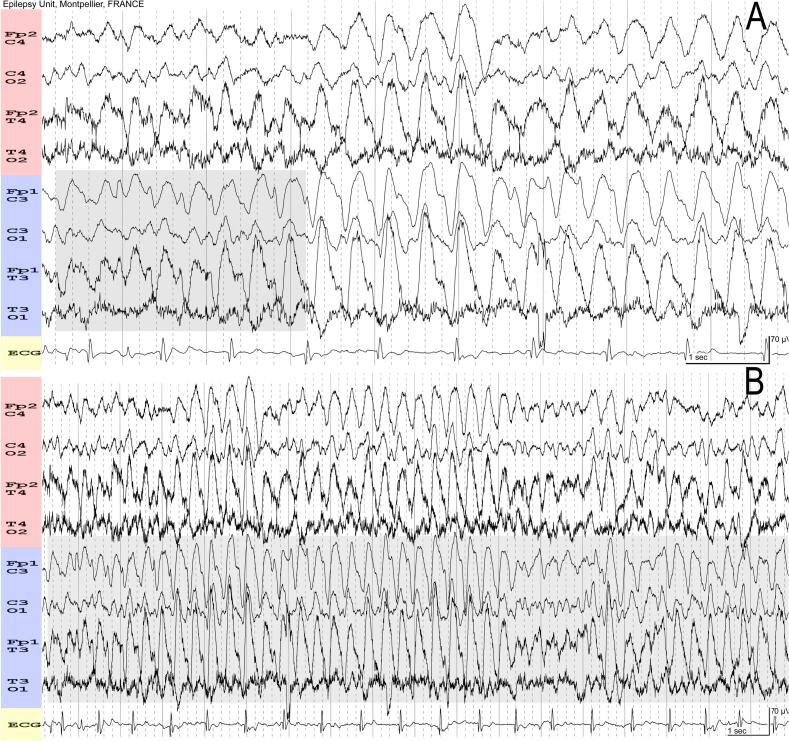
**A:** 30 mm/s, 7 µV/cm. An auditory stimulation provokes an arousal reaction with high-amplitude hypersynchronous delta waves characterized by asymmetrical, frontally predominant waves at 2.3 Hz (23 waves/10 s). These sharply contoured rhythmic delta waves (LRDA + S) are predominantly observed in the left hemisphere starting in the left frontal region (grey area) then spreading two seconds later to the right frontal region. Note the presence of muscle artifacts representing arousal. **B:** Same EEG panel at 15 mm/s and 7 µV/cm showing the full arousal response. Note the predominance of the discharge over the left hemisphere (grey area), consistent with a breach rhythm. This delta activity persists for over 10 s. The record was interpreted as having a stimulus-induced seizure, and the patient was considered to have nonconvulsive status epilepticus.

A 17-year-old male was admitted to the ICU following severe brain trauma that resulted in a depressed skull fracture on his left side. After undergoing neurointensive treatment and being kept in a medically induced coma, an EEG was conducted because the patient did not wake up when sedation was discontinued. The EEG results showed the presence of SI-discharges, which were characterized by asymmetrical rhythmic, frontally predominant delta waves at 2.3 Hz (23 waves/10 s) following stimulation. Sharply contoured delta activity, consistent with LRDA + S, was predominant in the left hemisphere. It originated in the left frontal region and, approximately 2 s later, propagated to the right frontal region (Fig. 1). The runs lasted longer than 10 s and demonstrated amplitude variation, meeting the criteria for an electrographic seizure.

**Fig. 2:**
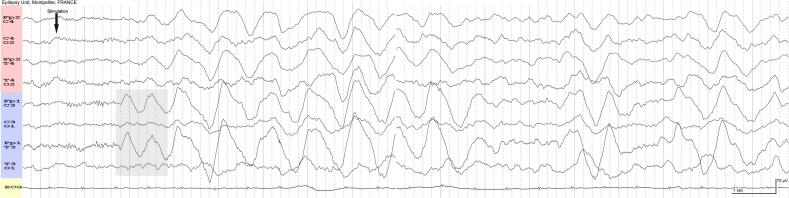
48 h later, sedated patient. An auditory stimulus (arrow) produces bilateral bursts of rhythmic delta waves at 1 Hz without any muscle artifact. The slow waves originate in the left frontal region (grey area), exhibiting higher amplitude over the left hemisphere. Repeat EEGs over the following days were similar, showing bilateral rhythmic delta activity after each stimulation. This activity then disappeared after IV clonazepam, along with the appearance of sleep patterns. The EEGs were reviewed and reinterpreted as representing an arousal reaction in a young adult.

Based on the EEG findings, with evolution in location but also in morphology and discharge duration (more than 10 s), the patient was diagnosed with focal subclinical seizures originating from the left hemisphere, and antiseizure medications (ASMs) were administered. Sedation was reinstated. SI-rhythmic delta activities were again observed during the decrease in sedation. Subsequent EEGs showed bilateral, slightly asymmetric sinusoidal delta waves with higher amplitude over the left hemisphere, occurring after sensory stimuli (Fig. 2). These delta waves demonstrated reactivity to IV clonazepam (CNZ), resulting in improved EEG. Although no clinical improvement was observed, the patient returned to sleep immediately. “Possible” NCSE was suggested as a potential cause of the delayed awakening, and sedation was maintained. The EEGs were reviewed and reinterpreted as representing an arousal reaction during the awakening process in a young adult. Withdrawal of the anesthetic drugs did not provoke any seizures, and his recovery was smooth. ASMs were progressively stopped. The patient was then transferred to a rehabilitation center for right hemiparesis.

## 3 Discussion

In comatose patients, NCSE can negatively impact awakening. An EEG is crucial for identifying seizures; interpretation, however, requires expertise. A pattern that consistently shows greater voltage in one hemisphere while also showing it in the other is classified as LRDA. This is in contrast to GRDA, which must be synchronous and symmetric between the two hemispheres (Hirsch et al., 2023, Hirsch et al., 2021). The clinical significance of this distinction is underscored by LRDA's link to an increased risk of seizures with frequency-dependent associations (Rodriguez Ruiz et al., 2017, Snider et al., 2023). In cases of definite evolution, LRDA may be considered ictal by itself (in terms of frequency changes, morphology, or spatial distribution over time) when the discharge lasts more than 10 s overall (Hirsch et al., 2023, Hirsch et al., 2021). In our case, the first EEG showed a definite evolution of LRDA + S, beginning in the left frontal region and spreading to the right (Fig. 1). The activity lasted for more than 10 s, which incorrectly classifies this activity as an electrographic seizure instead of an awakening reaction.

Awakening/Postarousal hypersynchrony, sometimes called hypnopompic hypersynchrony, is characterized by the appearance of bilateral rhythmic sinusoidal delta activity on the EEG. This normal awakening reaction is frequently seen in young children and declines from age five onward, but it may be observed in adolescents or young adults (Gélisse and Crespel, 2019). Patients with parasomnias, such as somnambulism, confusional arousal, and night terrors, experience this type of reaction more frequently, exhibiting bilateral hypersynchronous delta activity (Pressman, 2004). In the reported case, the morphology of this awakening hypersynchrony was unusual due to a clear predominance over the left hemisphere (Fig. 1). This asymmetry, with higher amplitude of the delta waves on the left hemisphere, corresponded to a breach rhythm (left-sided depressed skull fracture). The strict application of criteria for electrographic seizures, without taking into account the clinical context of an awakening reaction in an adolescent with a skull fracture history, led to an incorrect diagnosis of epileptic seizures.

The use of IV CNZ injections successfully stopped rhythmic delta activity, although without clinical improvement. According to the Salzburg criteria for NCSE, improvement of the EEG without clinical improvement after the administration of IV ASMs is labeled as “possible NCSE” (Leitinger et al., 2016), which carries a significant risk of overtreating patients with unnecessary medications. There is a common mistake in interpreting improvement of the EEG as proof that this activity was ictal (or possibly ictal) when the hypnotic effect of the drug is not taken into account (Gélisse and Crespel, 2025, Gelisse et al., 2021b). The effect of IV-CNZ injections in this patient was only related to the hypnotic effect of the drug, not its antiseizure effect. Postarousal hypersynchrony disappeared after IV-CNZ injections because the patient immediately returned to sleep.

## 4 Conclusion

Definitions and recommendations for EEG interpretation are useful, but it is important not to become restricted by these definitions. A holistic approach to the patient is required for EEG interpretation. In comatose patients, physiological awakening reactions must be examined. In the ICU context, problems with EEG interpretation may also arise in the presence of activity following stimulations, for which EEG interpreters use the acronym SIRPIDs. This acronym has become extremely popular, as it has enjoyed widespread use in the EEG literature and represents a concept that is easy to understand. Due to the ambiguity of the term “ictal” and its clinical implications, it could be substituted with “intermittent”: Stimulus-Induced Rhythmic or Periodic Intermittent Discharges**.** SIRPIDs are only transiently seen, occurring with each auditory or somatosensory stimulation either in the same EEG recording or during subsequent EEGs (Gélisse and Crespel, 2025, Gelisse et al., 2021a).

**Ethical Publication Statement**.

We confirm that we have read the Journal’s position on issues involved in ethical publication and affirm that this report is consistent with those guidelines.

## CRediT authorship contribution statement

**Philippe GELISSE:** Conceptualization, Writing – original draft, Writing – review & editing. **Arielle CRESPEL:** Writing – review & editing.

## Declaration of competing interest

**Dr. Gélisse** received support for teaching programs from UCB, Eisai and royalties for publishing from John Libbey Eurotext. **Dr. Crespel** received support for teaching programs from UCB, Eisai and royalties for publishing from John Libbey Eurotext.
